# The Food Contaminant Mycotoxin Deoxynivalenol Inhibits the Swallowing Reflex in Anaesthetized Rats

**DOI:** 10.1371/journal.pone.0133355

**Published:** 2015-07-20

**Authors:** Anne Abysique, Catherine Tardivel, Jean-Denis Troadec, Bernadette Félix

**Affiliations:** 1 EA 4674, Laboratoire de Physiologie et Physiopathologie du Système Nerveux Somato-Moteur et Neurovégétatif, Aix-Marseille University, Marseille, France; 2 INRA U1189, Département AlimH, St Genès Champanelle, France; University of California, Los Angeles, UNITED STATES

## Abstract

Deoxynivalenol (DON), one of the most abundant mycotoxins found on cereals, is known to be implicated in acute and chronic illnesses in both humans and animals. Among the symptoms, anorexia, reduction of weight gain and decreased nutrition efficiency were described, but the mechanisms underlying these effects on feeding behavior are not yet totally understood. Swallowing is a major motor component of ingestive behavior which allows the propulsion of the alimentary bolus from the mouth to the esophagus. To better understand DON effects on ingestive behaviour, we have studied its effects on rhythmic swallowing in the rat, after intravenous and central administration. Repetitive electrical stimulation of the superior laryngeal nerve or of the tractus solitarius, induces rhythmic swallowing that can be recorded using electromyographic electrodes inserted in sublingual muscles. Here we provide the first demonstration that, after intravenous and central administration, DON strongly inhibits the swallowing reflex with a short latency and in a dose dependent manner. Moreover, using c-Fos staining, a strong neuronal activation was observed in the solitary tract nucleus which contains the central pattern generator of swallowing and in the area postrema after DON intravenous injection. Our data show that DON modifies swallowing and interferes with central neuronal networks dedicated to food intake regulation.

## Introduction

Mycotoxins are toxic fungal metabolites that contaminate a wide range of cereals and cereal-derived products. Field fungi, such as the *Fusarium* species, produce mycotoxins on growing cereals, but also during crop storage after harvesting [1]. Produced by various *Fusarium* fungi species, Deoxynivalenol (DON) is the most abundant type B trichothecene found in cereals such as wheat, maize, barley, oats, rye, and less often rice and banana [2]. DON is characterized by a high stability under different environmental conditions and resistance to high temperature [3] which explains its widespread presence in human food. In this context, DON constitutes the most common contaminant of food stuffs [4–8]. The consumption of DON-contaminated cereals or cereal-derived products induces both acute and chronic toxic effets. In both humans and farm animals, acute exposure to high doses causes nausea, vomiting, and drowsiness, and may result in diarrhea, leukocytosis, gastrointestinal hemorrhage and ultimately death [9, 10]. Its capacity to induce vomiting episodes in various species including humans explains its commonly used nickname “vomitoxin” [11]. Exposure to moderate doses of this toxin induces anorexia, reduced weight gain and altered nutritional efficiency [12]. Recent studies have shown that DON reduces food intake and modifies satiation and satiety [13, 14]. This anorectic action seems to imply a DON-induced release of the satiety gut hormone PYY [15] and the activation of neuronal networks implicated in feeding behavior termination [14].

Swallowing is a major motor component of ingestive behavior, which allows the propulsion of the alimentary bolus along the gut. These motor processes require the coordination of various muscles localized in the mouth, larynx, pharynx and oesophagus. By propulsing food from the oral cavity into the stomach *via* the pharynx and the oesophagus, this complex reflex constitutes the first step of feeding behavior. The sensory afferent fibers convey through the superior laryngeal nerve (SLN), a branch of the vagus nerve, and project to the brainstem *via* the solitary tract (ST) at the dorsal vagal complex (DVC) level. The afferent fibers, which play an important role in triggering swallowing, contact premotoneurons mainly located within the interstitial and intermediate subnuclei of the solitary tract nucleus (NTS) in the brainstem. These premotoneurons, which modulate the motoneurons, constitute the central pattern generator of swallowing (SwCPG) [16]. Various neurotransmitter systems are involved in swallowing [17]. The main data concern the induction and facilitation of SwCPG activity by excitatory amino acids [18, 19], inhibition of the swallowing sequence by gaba amino butyric acid (GABA) [20, 21], and the modulatory influence of monoamines [22]. In previous studies, we have shown that the swallowing reflex is inhibited by anorexigenic substances such as leptin [23] and brain derived neurotrophic factor (BDNF) [24]. Recently, Mostafeezur and al. [25] reported that orexigenic cannabinoids facilitate the swallowing reflex. In this context, we hypothesized that DON, which exerts a potent anorectic action, may regulate the swallowing reflex. Based on this hypothesis, we evaluated the effect of intravenously (i.v.) or centrally administered DON, on the swallowing reflex elicited by electrical stimulation of either the peripheral SLN or the central ending of the afferent fibers localized in the ST. In both cases, the stimulation implied the afferent fibers involved in the rhythmic swallowing reflex. Further, to determine the central site of DON action, we investigated c-Fos expression induced by the mycotoxin.

In the present study, we demonstrated that DON inhibits the swallowing reflex shortly after its intravenous (i.v.) administration. A strong neuronal activation in the NTS and the area postrema (AP), attested by an increased c-Fos expression, was also observed after i.v. injection. Finally, the direct injection of DON within the SwCPG reduced rhythmic swallowing pattern.

## Materials and Methods

The experimental procedures described here were carried out in accordance with the directives of the French Ministry of Agriculture and Fisheries and the European Community Council (86/609/EEC). The protocol was approved by the committee on the ethics of animal experiments of Marseille/N°14 (authorization number: 01289.02).

### 1) Surgical procedures

Experiments were performed on 54 adult male Wistar rats weighing 350 g (Charles River, l’Arbresle, France), anesthetized with 0.6ml of a mixture of ketamine (100 mg/ml; Centravet, Dinan, France) and xylazine (15 mg/ml; Centravet, Dinan, France), injected intraperitoneally in a proportion of 90% and 10%, respectively. The anaesthesia was then continued by perfusion of the same mixture diluted at 10%, through a catheter inserted in the femoral vein, at a rate of 0.01–0.05 ml/h.

Under supine position, the superior laryngeal nerve (SLN) was dissected free from surrounding tissues and placed on miniature bipolar electrodes included in a plexiglas gutter. This device was sutured to the adjacent muscles, then the cutaneous planes were sutured to well maintain the stimulating device when the rat was fixed in a stereotaxic frame (Horsley and Clarke apparatus adapted for rats) under prone position. After occipitoparietal craniotomy and removal of the posterior part of the cerebellum, the floor of the fourth ventricle appeared to lie in a horizontal plane. The surface of the medulla was exposed in order to allow the stereotaxic introduction of the microelectrode in the NTS for drugs injection, and of the stimulatory bipolar electrode into the ST. The medulla was covered with warm liquid paraffin.

### 2) Experimental procedures for DON injections

**Fig 1:**
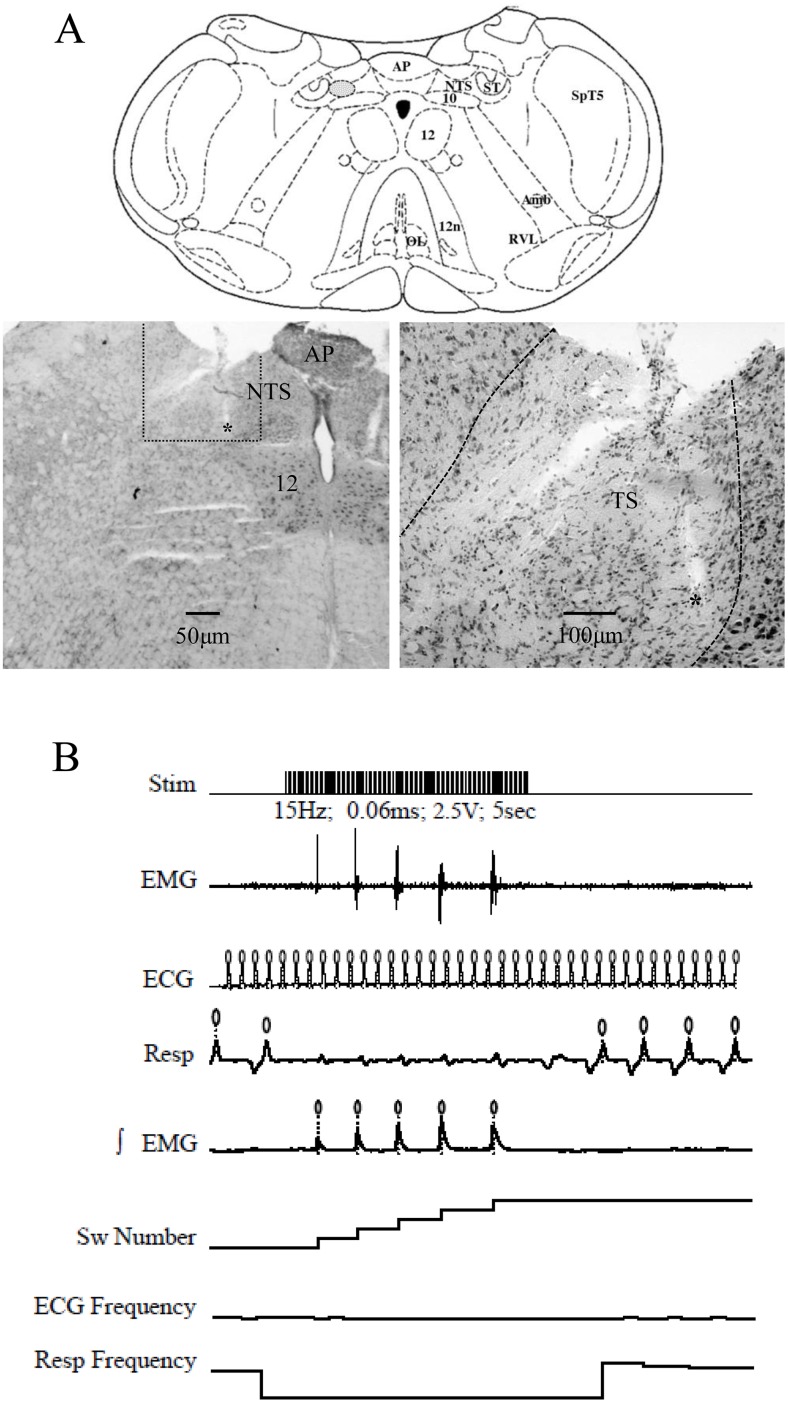
A. Schematic representation of the brainstem at the DVC level showing the central pattern generator of swallowing (SwCPG) and the solitary tract (ST). Coronal diagram of the medulla drawn from the atlas of Paxinos and Watson [27] corresponding to the level of glutamate and DON injection sites (between 0.2 and 0.5 mm in front of the caudal edge of the area postrema and 0.5 to 0.7 mm lateral) and to the level of stimulation of the solitary tract (ST). Although microinjections and ST stimulations were made on either side of the medulla, all injection sites have been reported to the left side and dotted in the hatched area corresponding to the region of the interstitial and intermediate solitary tract nucleus (NTS) subnuclei. Histological micrographs illustrating the stimulating ST site show the bipolar electrode tract (see *). Amb, ambiguous nucleus; AP, area postrema; OL, inferior olive; RVL, rostroventral reticular nucleus; SpT5, spinal trigeminal tract; ST, solitary tract; 10, dorsal motor nucleus of the vagus; 12, hypoglossal nucleus; 12n, hypoglossal nerve. B. Parameters recorded in the rat during the experiment. Stim, stimulation of the superior laryngeal nerve (SLN), at 15 pulses/s during 5 s (pulse parameters: 2.5V and 0.06ms); EMG, sublingual muscle electromyogram (note rhythmic swallowing triggered by SLN stimulation); ECG, electrocardiological activity; Resp, respiration (note respiration blockade during SLN stimulation), ∫EMG, electromyogram envelope signal normalized, Sw Number, number of swallows triggered by SLN stimulation; ECG Frequency and Resp Frequency; “o” above the recording are event markers for ECG, Resp and EMG. Note that ST stimulation produced similar responses.

We studied how DON affects the rhythmic swallowing elicited by repetitive stimulations of the SLN (or the ST). Previous studies have shown that a microinjection of glutamate within the intermediate NTS, containing the SwCPG, can initiate swallowing [18, 23, 26]. Thus, glutamate microinjection (1 fmol) was used as a control to check that the microelectrode was positioned within the SwCPG. When such a glutamate microinjection elicited swallowing, the stereotaxic coordinates were conserved for DON injections. The precise coordinates extended [23] between 0.2–0.7 mm rostral to the caudal edge of the AP (taken as the 0) 0.5–0.7 mm laterally and 0.4–0.8 mm in depth, roughly corresponding to those previously used. Fig 1A represents the precise area of drug injections (from Paxinos and Watson [27]).

DON obtained from Sigma (Saint Quentin Fallavier, France) was dissolved in NaCl 0.9% solution and delivered: *i*) in the SwCPG by pressure ejections through glass pipettes (70 μm O.D. at the tip) using an injection device (PMI-200, Dagan Corp., Minneapoli, MN USA). The pressure ejection was adjusted between 150 and 200 kPa for pulses of 3 seconds in duration, and the injected volume was 100 nl. *ii*) in the femoral vein *via* a polyethylene catheter. The injected volume was 500μl.

### 3) Stimulations and recordings

Swallowing was triggered by two different ways: *i*) peripheral stimulation of the sensitive fibers contained in the SLN *ii*) central stimulation of the solitary tract (ST) corresponding to the entering of the sensitive fibers in the brainstem. The main way to induce swallowing was SLN stimulation. However, during the experiment, SLN stimulation could become less efficient. To avoid increasing too much stimulation parameters, the ST stimulation (Fig 1A) was applied. The bipolar electrode was lowered into the ST using stereotaxic coordinates, and an electrical current was applied with parameters (voltage, duration) adjusted to that of the preceding SLN stimulation until the rhythmic swallowing was induced. SLN and ST stimulation concerned the same afferent fibers involved in the swallowing reflex.

Stimulation with a long train of pulses produced several swallows [or rhythmic swallowing recorded by electromyography (EMG)], at a rhythm depending on stimulation frequency that constitutes a specific pattern of swallowing. In the present study, repetitive long trains of pulses (5 s duration at 5–30 Hz frequency every 30 s) were used. The pulse voltage, duration and frequency varied according to the animal (1.5–5 V; 0.02–0.5 ms) in such a way as to trigger around 4–6 rhythmic swallows. To monitor swallowing, the EMG activity of sublingual muscles (mainly the geniohyoïd) was recorded by means of bipolar copper wire electrodes inserted into the muscles, using a hypodermic needle. The respiratory activity was recorded by means of a mechanotransducer placed around the thorax, and the electrocardiogram (ECG) by subcutaneous electrodes placed on each side of the thorax. Moreover, the electrocardiogram and swallowing EMG signals were fed to loud speakers for auditory monitoring. Rectal temperature was monitored and maintained around 37°C with a heating pad. The EMG, ECG and respiration signals were recorded on a computer using an analog-to-digital interface (PowerLab 8SP data acquisition software for Windows, ADInstruments, USA).

### 4) Signal Analysis

Either peripheral or central stimulations of afferent fibers triggered reflex swallowing. As an example, repetitive electrical stimulation (every 30 s) of the SLN (2.5 V; 0.06 ms; 15 Hz, 5 s) elicited rhythmic swallowing (Fig 1B). A stable control sequence involving three 5s trains of stimulations was performed before DON injection. The mean values obtained during this sequence were used as control values. Afterwards, stimulations and recordings were maintained until recovery.

The SLN or ST stimulation parameters produced a powerful cessation of respiration, which accompanied rhythmic swallowing. A specially designed computer program Chart5.5 software calculated: ∫EMG (electromyogram envelope signal normalized), Sw Number (number of swallows triggered by SLN or ST stimulation), ECG and respiratory frequency.

### 5) Immunohistochemistry

#### PBS perfusion and immersion-fixation

Animals pretreated by DON (3mg/kg, iv, n = 5) or by NaCl (0.9%, iv, n = 5) were used for c-Fos immunostaining. Two hours after injection, rats under deep anesthesia, were perfused intracardially with 250ml of ice-cold 0.1M phosphate buffered saline (PBS, pH 7.4). Rats were decapitated immediately; thereafter the brain extracted from the skull was immersed into ice-cold freshly prepared solution of 4% paraformaldehyde in 0.1M phosphate buffer (PB) overnight. Brains were rinsed in PBS and cryoprotected for 24–48h in 30% sucrose at 4°C. The brains were frozen in isopentane (-40°C), then coronal sections (40μm thick) of the brainstem were realized with a cryostat (Leica CM3050, France) and collected serially in 0.1M PBS.

#### c-Fos immunohistochemistry

Brainstem sections were incubated for 10 minutes in 0.1M PBS containing 1.5% H_2_O_2_ for quenching of endogenous peroxidase activity. After one hour in saturation PBS buffer containing 3% normal goat serum and 0.3% triton X-100, sections were incubated for 48h at 4°C with a rabbit anti-c-Fos antibody (1:10.000 Ab-5, Calbiochem). A biotinylated goat anti-rabbit IgG (1:500, Vector Labs) was used as a secondary antibody (incubated for 2h at room temperature). Peroxidase activity was revealed using the avidin-biotin complex (1:200, Vector Labs) and diaminobenzidine as chromogen. Non-specific labelling was observed on adjacent sections that were treated identically but without the primary antibody. The reaction was closely monitored and terminated by washing the sections in distilled water when optimum intensity was obtained (3–5 min).

#### Image analysis and c-Fos positive neuron count

For each animal, c-Fos immunostaining analysis was performed by counting the positive nuclei on four non-consecutive sections using microphotographs acquired by a 10 fold lens with a DMX 1200 camera (Nikon) coupled to ACT-1 software. The microscope was set at a specific illumination level, as was the camera exposure time. c-Fos positive nuclei were then counted on these pictures by computer-assisted morphometry using the ImageJ software as previously described by Girardet et al. [14].

### 6) Statistical analyses

All swallows calculated values were normalized as percents of control values. Statistical analyses were performed using one- or two- way analysis of variance (ANOVA) followed by Fisher’s protected least-significant difference post-hoc test for all electrophysiological data, and using Mann-Whitney test for immunohistological data (StatView for Windows 5.0.1; SAS Institute). Differences were considered significant when P < 0.05. Data were expressed as mean ± SEM.

## Results

The results presented in this study come from the analysis of raw data summarized in S1 Table.

### DON administered intravenously inhibited swallowing

**Fig 2:**
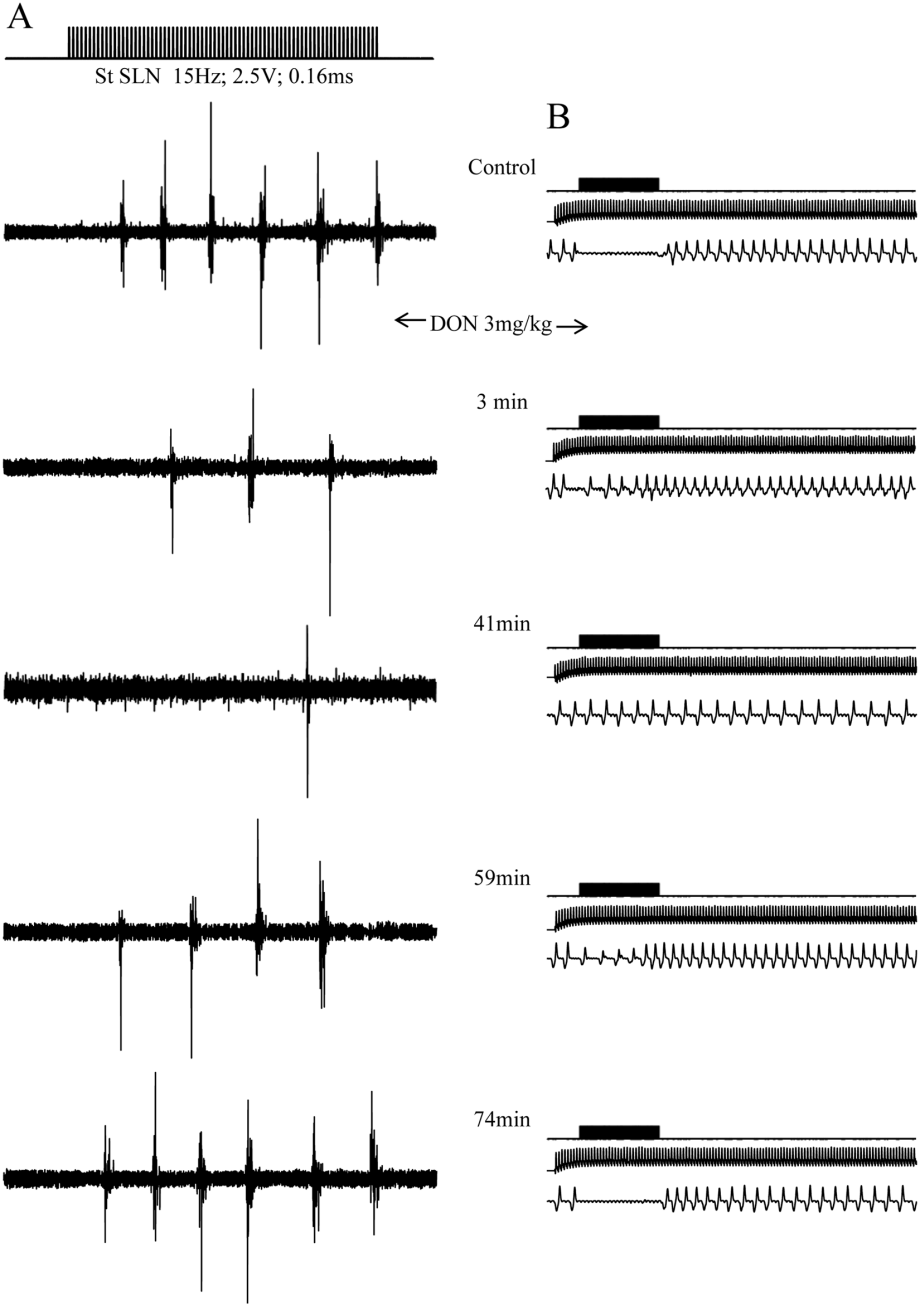
Intravenously injected DON inhibited rhythmic swallowing triggered by SLN stimulation. A. Typical sublingual muscle electromyogram (note the rhythmic swallowing induced by SLN stimulation). DON (3mg/kg bw) injection induced a rapid and powerful inhibition of the number of swallows. 3 minutes after DON application, only three swallow were triggered by SLN stimulation. B. Electrocardiogram and respiratory activity recorded before, during and after SLN stimulation. Note the blockade of respiration induced by swallowing during SLN stimulation in control condition and at recovery 74 min after DON injection.

**Fig 3:**
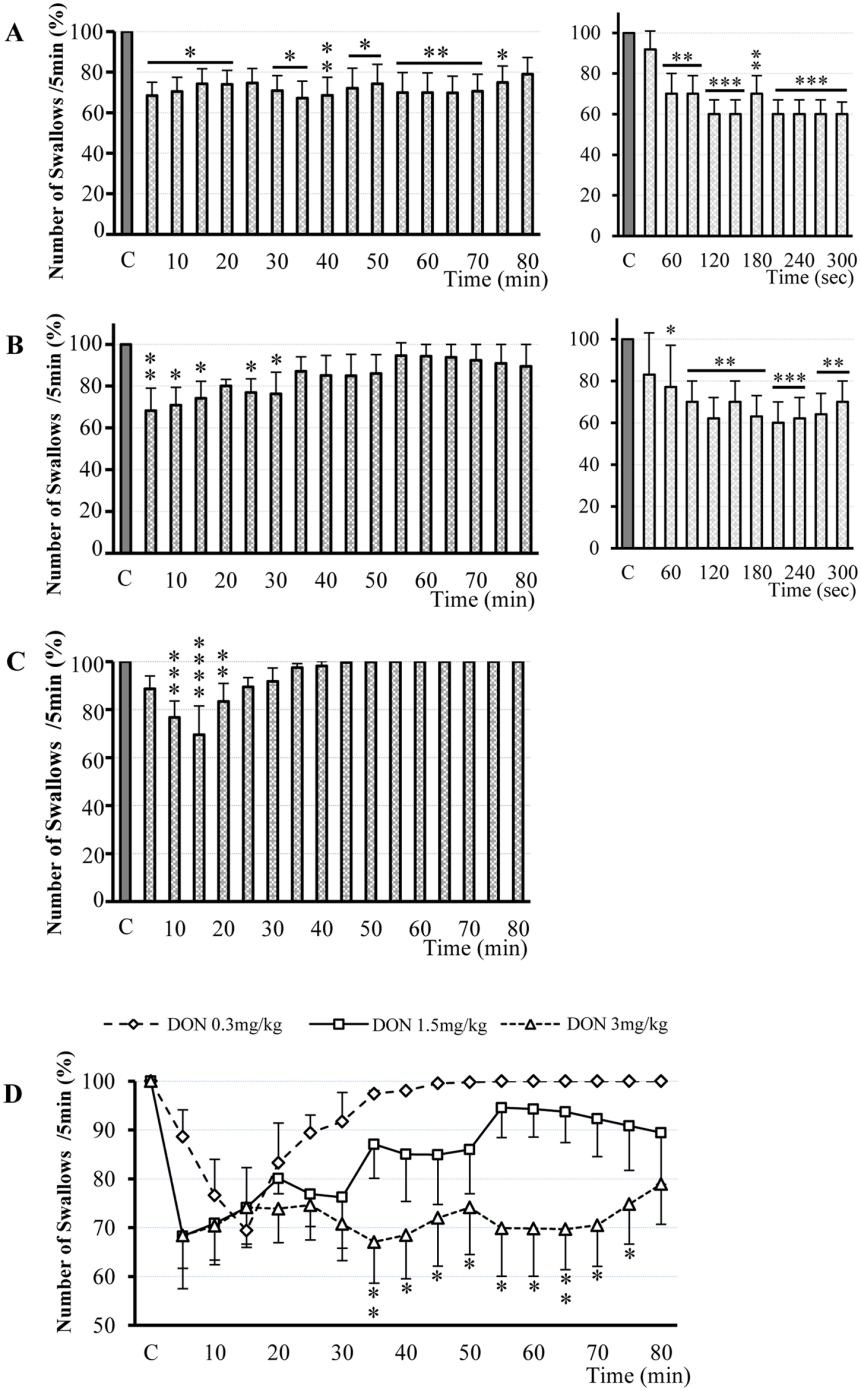
Dose-dependent DON inhibition of rhythmic swallowing triggered by SLN or ST stimulation. Time course of the effect of various DON doses intravenously injected: 3mg/kg bw (16 trails; A), 1.5mg/kg bw (7 trials, B) and 0.3mg/kg bw (9 trials; C) on the number of swallows recorded over 5 minute periods and until recovery. On the right side of A and B, note a focus on the kinetics of DON effect over the first 5 min. Black bars represent the mean value of the control recorded before DON injection (C, 100%). Data are represented as means ± SEM normalized to the control value. D: Comparison of the inhibitory effects of the three doses of DON on rhythmic swallowing triggered by SLN or ST stimulation. Note that time course variations according to the doses showed a significant inhibitory effect for DON 3mg/kg bw, compared with the other doses, beginning 35 minutes after injection and lasting 40 minutes. *P<0.05, ** P<0.01, *** P<0.001, **** P<0.0001.

Effects of DON were studied after intravenous administration on 24 rats from 32 trials. All doses of DON induced a significant inhibition of the swallows recorded during SLN or ST stimulation. At 3mg/kg body weight (bw) (14 rats, 16 trials) DON decreased the number of swallows with a latency of 2.91 ± 1.45 min [Figs 2A and 3A, Table 1]. This effect was maximal between 40 and 70 minutes after DON injection (32.1 ± 8.6 to 29.5 ± 8.4% decrease, P < 0.01) and lasted 70.61 ± 4.43 min. At 1.5 mg/kg bw (4 rats, 7 trials), DON induced a significant decrease in the number of swallows triggered by SLN or ST stimulation after a latency of 1.36 ± 0.42 min (Fig 3B and Table 1). Compared with the 3mg/kg dose, the effect presented similar amplitude (31.7 ± 10.7% decrease, P < 0.01) but a shorter duration, 41.43 ± 9.20 min. In the 6 tested rats (9 trials), injections of 0.3mg/kg bw DON decreased significantly the number of swallows 7 ± 1 min after the injection onset (23.4 ± 7.0%; P < 0.001) and this effect lasted 25.83 ± 7.64 min (Fig 3C and Table 1). Statistical comparison of the inhibitory effects of DON doses (Fig 3D) showed that inhibition time course was significantly different only between 3mg/kg and 0.3mg/kg. The difference between the two doses became significant between 35 and 75 minutes after DON injection.

**Table 1:** Effects of DON injection on the real number of swallows triggered by SLN (or ST) stimulation.

**i.v. DON dose**	**Control Value**	**After DON injection**
3 mg/kg	4.9 ± 0.1	2.9 ± 0.4 (after 2min)
1.5 mg/kg	4.5 ± 0.2	2.8 ± 0.6 (after 2min)
0.3 mg/kg	4.1 ± 0.2	2.9 ± 0.4 (after 15min)
**SwCPG DON dose**	**Control Value**	**30s after DON injection**
570 ng/kg	4.1 ± 0.3	1.2 ± 0.5
150 ng/kg	5.4 ± 0.2	2.3 ± 0.6
60 ng/kg	4.7 ± 0.2	3.7 ± 0.5

The real number of swallows recorded is indicated as mean ± sem. For each dose of DON injected, mean values of swallows recorded at a time when a robust inhibitory effect of swallows was noted; except for 60ng/kg DON dose which did not modify the number of swallows. i.v., intravenous administration; SwCPG, central microinjection.

All doses of DON administered intravenously inhibited the rhythmic swallowing pattern, without any variation of either cardiac frequency or respiratory activity (32 trials; 24 rats) (Fig 2B).

### Effects of intravenous injection of DON on c-Fos expression in the brainstem

**Fig 4:**
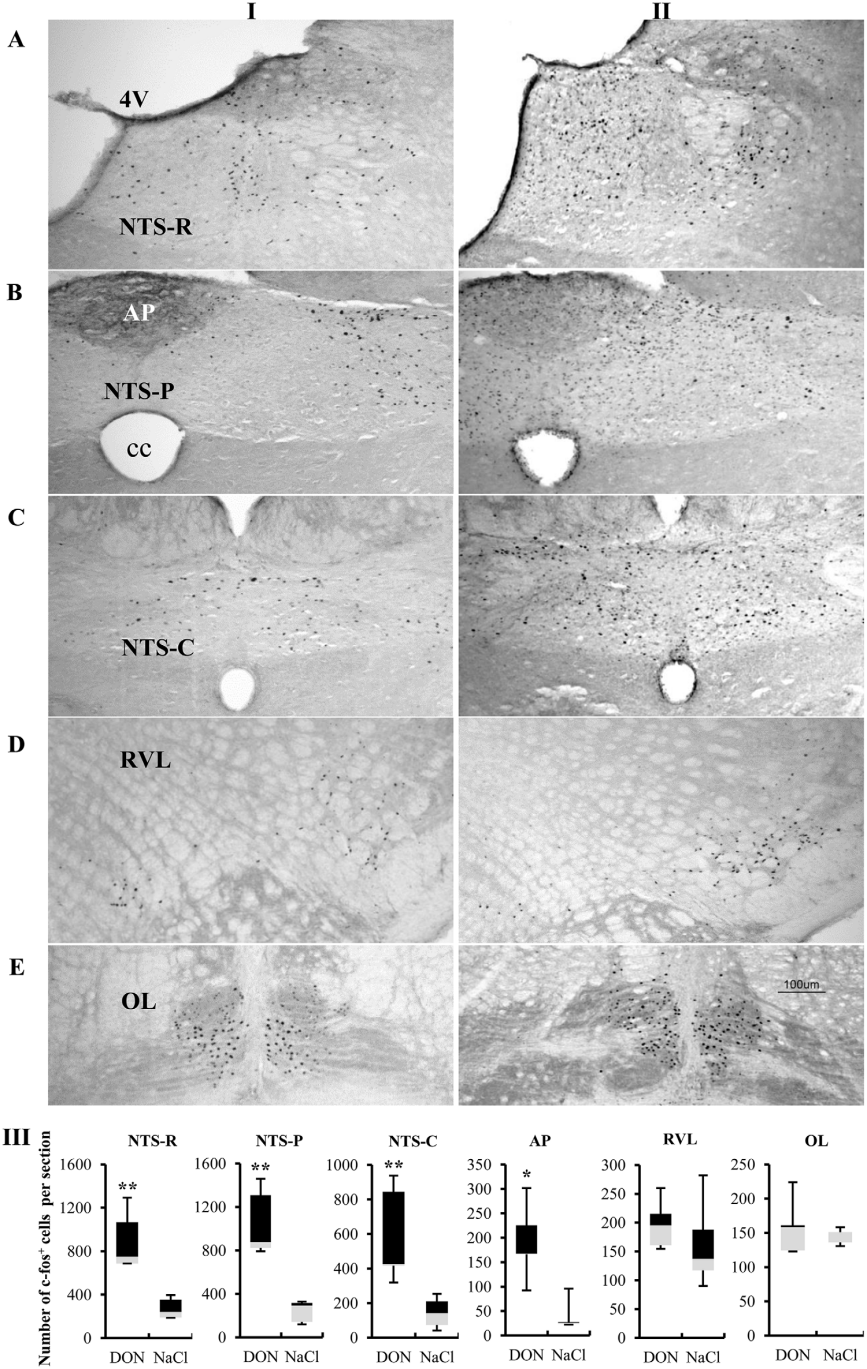
Effects of DON intravenous administration on c-Fos immunoreactivity. Representative coronal sections illustrating the c-Fos labeling observed in the brainstem (A, B, C: NTS, D: RVL, E: OL) of I) control rats intravenously treated with NaCl (0.9%) and sacrificed 2h post-treatment, II) rats intravenously treated with DON (3mg/kg bw) and sacrificed 2h post-treatment. III) Quantification of the number of c-Fos immunoreactive nuclei in the brainstem observed in rats treated either with DON (3mg/kg bw) or NaCl (0.9%). *P<0.05, **P<0.01 significantly different from NaCl-treated rats. Box explanation: upper horizontal line of box, 3^rd^ quartile; lower horizontal line of box, 1^st^ quartile; color separation within box, median; upper horizontal bar outside box, max; lower horizontal bar outside box, min. 4V, fourth ventricle; AP, area postrema; cc, central canal; NTS-C, caudal part of the NTS; NTS-R, rostral part of the NTS; NTS-P, postremal division of the NTS; OL, inferior olive; RVL, rostroventral reticular nucleus. Scale bar: 100 μm.

Central structures activated in response to intravenous administration of DON were identified using the immune detection of the c-Fos protein. A low basal level of c-Fos positive nuclei was observed in the brainstem of control (NaCl) rats (Fig 4I). DON-treated rats exhibited a significant increases in the number of c-Fos positive nuclei throughout the dorsal vagal complex (DVC) including the nucleus tractus solitarius (NTS) and the area postrema (AP) (Fig 4II). Counts of positive nuclei in the DVC revealed significant increases in the number of c-Fos labeled nuclei in treated animals compared with control animals. In DON-treated rats, we observed a strong c-Fos induction in four sub-regions of the DVC: rostral part, postremal division and caudal part of the NTS, and the AP (Fig 4AII, 4BII and 4CII). In DON treated rats, c-Fos expression levels varied according to the sub-division of the DVC (Fig 4III): *i*) in the rostral NTS, the mean number of c-Fos positive nuclei per section reached 895.95 ± 121.5 *versus* 272.85 ± 43.21 in control rats. *ii*) in the postremal NTS, the number of c-Fos positive nuclei reached 1051.2 ± 72.16 *versus* 240.45 ± 30.9 in control rats. *iii*) in the caudal NTS, the number of c-Fos positive nuclei reached 588.5 ± 125.69 *versus* 143.85 ± 39.8. *iiii*) in the AP the number of c-Fos positive nuclei reached 190.5 ± 34.86 *versus* 38.75 ± 14.28 in control rats. The other nuclei positively stained, ie the rostroventral reticular nucleus (RVL) and the inferior olive (OL), exhibited no increase in c-Fos signals in response to DON administration (Fig 4DII, 4EII, and 4III).

### DON microinjections within the SwCPG inhibited swallowing in a dose dependent manner

**Fig 5:**
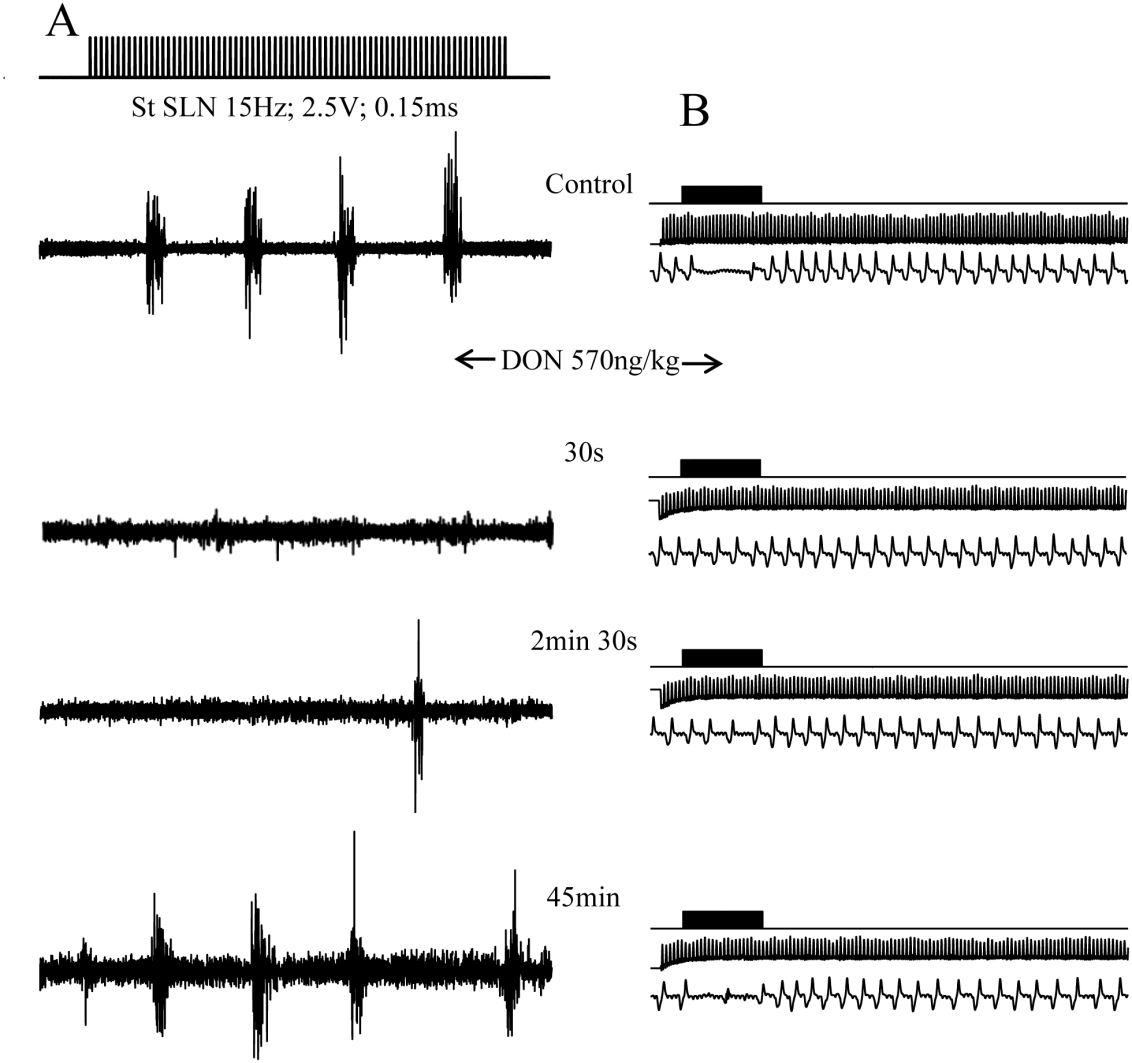
DON (570ng/kg bw; 10 trials) microinjection within the central pattern generator of swallowing (SwCPG) inhibited rhythmic swallowing triggered by SLN stimulation. A. Electromyogram from sublingual muscles (note the rhythmic swallowing induced by SLN stimulation). DON microinjection at 570ng/kg bw induced a rapid and powerful inhibition of the number of swallows. 30 seconds after DON application, no more swallow was triggered by SLN stimulation. This effect was transient since the swallows recovered after 45 minutes. B. Electrocardiogram and respiratory activity recorded during 30 seconds (before, during and after SLN stimulation). Note the blockade of respiration induced by swallowing during SLN stimulation in control condition and at recovery 45 min after DON injection.

**Fig 6:**
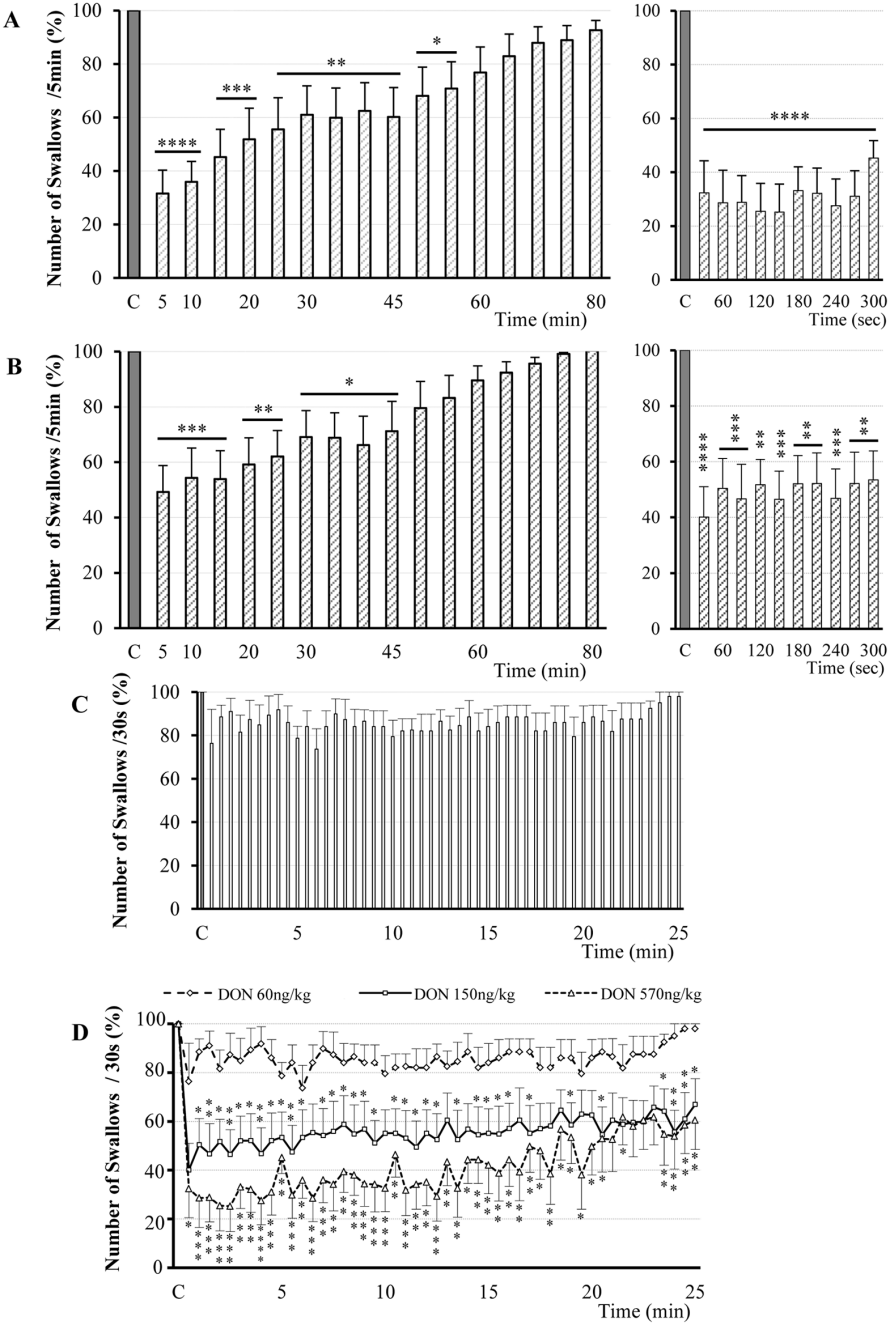
DON microinjection within the central pattern generator of swallowing (SwCPG) inhibited rhythmic swallowing triggered by SLN or ST stimulation in a dose depend manner. Time course of the effects of various doses of DON: 570ng/kg bw (10 trials; A), 150ng/kg bw (10 trials; B), 60ng/kg bw (10 trials; C) on the number of swallows recorded over 5 minute periods (for 570ng/kg bw and 150ng/kg bw) or over 30 second periods (for 60ng/kg bw). On the right side of A and B, note a focus on the kinetics of DON effect over the first 5 min. Black bars represent the mean value of the control recorded before DON injection (C, 100%). Data are represented as means ± SEM normalized to the control value. Note time course variations according to the doses showing a significant inhibitory effect immediately after DON microinjection for 570ng/kg bw and 150ng/kg bw. At 60ng/kg bw, DON microinjection did not modify rhythmic swallowing triggered by SLN stimulation. D: Comparison of the effects of the three doses of DON during the first 25 minutes after injection. Note the powerful effect of 570ng/kg bw and 150ng/kg bw DON doses. *P<0.05, ** P<0.01, *** P<0.001, **** P<0.0001.

The present results, obtained from 30 trials performed on 20 rats, showed that DON microinjections induced a significant dose-dependent decrease in the number of swallows recorded during SLN (or ST) stimulation. At the 570ng/kg dose (10 trials, 9 rats), DON induced immediately a significant powerful decrease of swallows and this inhibition persisted during 43.35 ± 9.71min [Figs 5A and 6A, Table 1]. The maximal effects of DON occurred after 6s and persisted during the 20 first minutes (68.4 ± 8.76% to 48.18 ± 11.5%, P < 0.0001 and P < 0.001). At the 150ng/kg dose (10 trials, 6 rats), DON immediately induced a significant powerful decrease of swallows and the inhibition persisted during 35.9 ± 4.63min [Fig 6B and Table 1]. The maximal effects occurred immediately after DON microinjections and persisted during 15 first minutes (60.8 ± 9.61% to 46.1 ± 10.3%, P < 0.001). In contrast, DON at the 60ng/kg dose (10 trials, 5 rats), failed to alter swallowing pattern characteristics, therefore this DON dose could be considered as subthreshold in our experimental conditions [Fig 6C and Table 1]. Statistical comparison of the inhibitory effects of DON doses during the first 25 minutes after injection, showed a significant dose-dependent effect (Fig 6D). DON injected within the SwCPG inhibited the rhythmic swallowing pattern, without any variation of either cardiac frequency or respiratory activity (30 trials; 20 rats) (Fig 5B).

## Discussion

The present study constitutes the first demonstration that DON mycotoxin inhibits the swallowing reflex. Acute intravenous (i.v.) injection of increasing DON doses (0.3, 1.5 or 3 mg/kg) transiently reduced the number of evoked swallows in a dose-dependent manner. The DON-induced effect on swallowing appeared to be specific, since inhibition was never observed with NaCl alone and DON modified neither heart rate nor respiratory rhythm. The doses of DON used here are classically employed to study acute intoxication in different models [28]. While oral toxin administration mimics an ingestion of DON-contaminated feed, in the present work, DON was intravenously administered on anaesthetized animals. Nonetheless, DON has been shown to cross the gut barrier after ingestion [29, 30] and is found in the systemic circulation after DON gavage [31, 32]. In our study, the duration of the inhibitory effect was dose-dependent and the highest dose reduced swallowing for more than one hour. By allowing the propulsion of the alimentary bolus along the gut, swallowing is a motor component of the ingestive behavior and a process critical for normal food intake [16]. Thus, our results strengthen the characterization of the DON effects on feeding behavior. DON ingestion induces reduction of food intake and even emesis in different animal models. Eating naturally contaminated food causes partial or complete feed refusal depending of the contamination degree [33, 34]. Previously, we had shown that anorexigenic factors, such as the adipose tissue-derived hormone leptin and the growth factor BDNF, inhibit the swallowing reflex [23, 24]. Inversely, Mostafeezur and al. [25] reported that orexigenic cannabinoids exert a facilitation of the swallowing reflex. Hence, the present data allow to classify DON among anorexigenic substances that both decrease food intake and inhibit swallowing. To be complete, it should be mentioned that this correlation between food intake and swallowing modulation is not quite as straightforward as it seems since orexigenic compounds including ghrelin [35] and orexin [36] were shown to inhibit the swallowing reflex.

One intriguing question is the mechanism by which intravenously injected DON inhibits the swallowing reflex. Some arguments support the hypothesis of DON-induced systemic increase in bioactive molecules that may act on peripheral targets. Among these putative effectors, serotonin (5-HT) and its derivatives could be envisaged. The modulatory influence of monoamines on the swallowing reflex is reported by several authors [16, 37] and 5-HT has well-known emetic and food intake reducing effects [38]. The serotoninergic system was also proposed to play a role in the triggering of feeding refusal, emesis and anorexia observed during DON intoxication [39]. However, it was recently reported that, in minks, an elevation in 5-HT plasma concentration followed rather than preceded the emetic activity. Indeed, plasma 5-HT elevation was only detectable 60 min after DON administration [40]. The kinetics of DON-induced inhibition of the swallowing reflex (<5 min) weakens the hypothesis of a possible involvement of peripherally released 5-HT. Otherwise, cytokines proinflammatory, which expression and release are increased by DON treatment may also be suggested [41]. These proinflammatory cytokines could act directly on striated muscular fibers involved in swallowing or by a reflex pathway to inhibit swallowing. Gaigé and al. [42] have shown, in the cat, that leptin enhances the intestinal electromyographic activity *via* a long loop reflex involving intestinal vagal afferent fibers with a release of Il-1ß. The superior laryngeal nerve being a branch of the vagus nerve, we can therefore hypothesize that proinflammatory cytokines released during DON intoxication could activate afferent fibers running in the SLN to change the activity of muscles involved in swallowing. However, as mentioned above, the kinetics of DON-induced swallowing inhibition seems to be poorly compatible with DON action on proinflammatory effectors. Even though oral exposure to DON was shown to increase the expression of proinflammatory cytokines, these acute stimulatory effects were observed 2 h after treatment [39, 41, 43–45].

Although much of the present discussion focuses on a peripheral action of DON and on the putative identity of peripheral effectors, it cannot be excluded that the toxin directly targets the central nervous system (CNS) to modulate the swallowing reflex. Consistent with a possible direct action of DON on the CNS, it was previously shown that the toxin is detectable in the brain of mice within 5min of oral exposure [44]. Furthermore, we have recently demonstrated that central DON injection mimics *per os* DON-induced anorexia [14]. In the present study, we showed that i.v. injection resulted in a strong and significant increase in c-Fos expression within the NTS and the AP. This DON-induced c-Fos expression was specific as it was not found in other brainstem nuclei i.e. RVL and OL, structures activated by the movement of striated muscles consecutive to the stimulation procedure. When DON was injected intravenously, its effects must have been larger than when injected in the SwCPG and in particular it may have acted on the entire NTS involved in various autonomic functions. C-Fos data show neuronal activation in various parts of the NTS involved in autonomic functions including respiratory and cardiac functions. However, electrophysiological data do not present any variations of respiratory and cardiac activities. The data suggest that these functions were not affected by the DON dose reaching the dedicated neurons. Moreover, in a previous study, Ngampongsa and al. [46] showed that DON administrated subcutaneously caused changes in heart rate but only 90 minutes after injection. In our study, after the highest dose of DON administered intravenously, swallowing was inhibited during 70 minutes and during these 70 minutes, we did not find any variation of cardiac or respiratory activities. The immunostaining observed in the AP is remarkable since this structure is a well-known pathway for systemic substances to reach the brain. Ossenkopp and al. [47] reported that area postrema ablation prevents the systemic DON-induced conditioned taste aversion in rats. These data suggest that the area postrema could be a gateway through which DON reaches the brain. Interestingly, Willis and al. [48] have shown that circulating compounds with a molecular weight about 0.3 kDa can diffuse from the area postrema to surrounding nuclei (i.e. NTS). It should be noted that the molecular weight of DON is 0.29 kDa. Moreover, the existence of vessels in the dorsomedial part of the NTS that lack immunoreactivity for the blood-brain barrier markers was reported [49]. These vessels may represent a route of entry for circulating substances to neurons in the NTS. All these data suggest that part of intravenously administered DON could act at the brain level. To investigate this assumption, we studied the effect of DON microinjections within the SwCPG. They induced a transient significant inhibition of rhythmic swallowing. This inhibitory effect appeared to be specific, since swallowing inhibition was never observed with NaCl alone. DON microinjection is not an intracellular injection and may concern both the passage of axons and NTS neurons surrounding the injection site. DON could indeed reach all these neural components but in our experimental protocol, only the swallowing reflex was affected. Finally, while the DVC is also an integrative center for autonomic cardiac and respiratory functions, the highest DON dose affecting swallowing had no effect on heart rate or respiratory frequency, confirming that DON specifically acts on ingestive functions rather than on other autonomic functions.

The cellular mechanisms through which DON could modify neuronal activity leading to an inhibition of the swallowing reflex are still unknown. Our study highlights the very short latency of DON-induced swallowing inhibition after its central microinjection. To our knowledge, this is the first description of such a rapid DON effect. To date, DON has been described as a ribosome poison that disturbs this organite and in turn inhibits protein synthesis [39]. In mice, *per os* DON administration inhibits protein synthesis in the spleen, peyer’s patches, kidney, liver, intestine and plasma from 3 h to 9 h post-treatment [43]. It was recently suggested that DON ribotoxic effect induces a modulation of mitogen activated protein kinases (MAPKs) activity. In turn, MAPKs activation may result in transcription factor activation that induces for instance cytokine or COX-2 expression [50–52]. It was also suggested that other signaling factors such as double stranded RNA-activated protein kinase or hematopoietic cell kinase relay DON toxicity from ribosome inhibition to MAPKs activation [53–55]. All these molecular mechanisms, which require minutes/hours delay to operate, appear poorly compatible with the DON action described here. Indeed, we showed that DON inhibits the swallowing reflex with a very short latency especially following central administration (<30 sec). It can be reasonably envisaged that DON might be able to induce rapid modulations of ionic conductance in neurons resulting in modifications of neuronal electrical activity. These alternative mechanisms were already proposed to explain toxicity of other mycotoxins. T-2 toxin has been shown to modify the activity of amino acid and glucose transporters, as well as calcium and potassium channels on myoblast cell membrane [56]. This toxin was also shown to increase cellular calcium concentration in the human promyelotic line HL-60 [57]. Whether such mechanisms may account for the DON-induced swallowing inhibition remains to be tested. Nevertheless, it appears from the present study that the electrophysiological recording of rhythmic swallowing constitutes a suitable model to investigate this hypothesis.

In summary, the present study demonstrates that the food contaminating toxin, Deoxynivalenol, inhibits the SwCPG in anesthetized rats and consequently the activity of motoneurons involved in swallowing. After i.v. administration, we observed a strong c-Fos expression within the NTS indicating an increased neuronal activity in this structure containing the SwCPG and also within the AP, specific area involved in the crossing of peripheral substances to the brain. Altogether, these data suggest an action of the toxin at the SwCPG level. Reinforcing this assumption, we observed a striking inhibitory effect after DON microinjection within the SwCPG.

Because this motor activity has a major role in ingestion, it can therefore be inferred that DON inhibits ingestion by acting not only on the afferent and integrative components of the structures involved in feeding but also on its motor outputs. The consequences of a DON-induced swallowing modulation should be considered on vulnerable and predisposed individuals suffering from dysphagia or swallowing impairment.

## Supporting Information

S1 Table The complete basic data on which this study was based. (XLSX)
